# Genetic Decomposition of the Heritable Component of Reported Childhood Maltreatment

**DOI:** 10.1016/j.bpsgos.2023.03.003

**Published:** 2023-03-24

**Authors:** Abigail R. ter Kuile, Christopher Hübel, Rosa Cheesman, Jonathan R.I. Coleman, Alicia J. Peel, Daniel F. Levey, Murray B. Stein, Joel Gelernter, Christopher Rayner, Thalia C. Eley, Gerome Breen

**Affiliations:** a Social, Genetic & Developmental Psychiatry Centre, Institute of Psychiatry, Psychology & Neuroscience, King’s College London, London, United Kingdom; b National Institute for Health and Care Research Maudsley Biomedical Research Centre at South London and Maudsley National Health Service Foundation Trust, London, United Kingdom; c National Centre for Register-based Research, Aarhus Business and Social Sciences, Aarhus University, Aarhus, Denmark; d PROMENTA Research Center, Department of Psychology, University of Oslo, Oslo, Norway; e Department of Psychiatry, Yale School of Medicine, New Haven, Connecticut; f Veterans Affairs Connecticut Healthcare System, West Haven, Connecticut; g Veterans Affairs San Diego Healthcare System, San Diego, California; h Department of Psychiatry and School of Public Health, University of California San Diego, La Jolla, California

**Keywords:** Childhood adversity, Childhood trauma, Explained variance, Genetic correlations, GWAS, Maltreatment

## Abstract

**Background:**

Decades of research have shown that environmental exposures, including self-reports of trauma, are partly heritable. Heritable characteristics may influence exposure to and interpretations of environmental factors. Identifying heritable factors associated with self-reported trauma could improve our understanding of vulnerability to exposure and the interpretation of life events.

**Methods:**

We used genome-wide association study summary statistics of childhood maltreatment, defined as reporting of abuse (emotional, sexual, and physical) and neglect (emotional and physical) (*N* = 185,414 participants). We calculated genetic correlations (*r*_g_) between reported childhood maltreatment and 576 traits to identify phenotypes that might explain the heritability of reported childhood maltreatment, retaining those with |*r*_g_| > 0.25. We specified multiple regression models using genomic structural equation modeling to detect residual genetic variance in childhood maltreatment after accounting for genetically correlated traits.

**Results:**

In 2 separate models, the shared genetic component of 12 health and behavioral traits and 7 psychiatric disorders accounted for 59% and 56% of heritability due to common genetic variants (single nucleotide polymorphism–based heritability [h^2^_SNP_]) of childhood maltreatment, respectively. Genetic influences on h^2^_SNP_ of childhood maltreatment were generally accounted for by a shared genetic component across traits. The exceptions to this were general risk tolerance, subjective well-being, posttraumatic stress disorder, and autism spectrum disorder, identified as independent contributors to h^2^_SNP_ of childhood maltreatment. These 4 traits alone were sufficient to explain 58% of h^2^_SNP_ of childhood maltreatment.

**Conclusions:**

We identified putative traits that reflect h^2^_SNP_ of childhood maltreatment. Elucidating the mechanisms underlying these associations may improve trauma prevention and posttraumatic intervention strategies.

SEE COMMENTARY ON PAGE 587

Traumatic events, namely, events perceived as physically or emotionally threatening and violating, are associated with various adverse outcomes, including psychopathology (1, 2, 3, 4). Behavioral genetics research conducted over decades has shown that reported trauma exposures, similar to many environmental measures and behavioral traits, are partly heritable (5,6). Twin studies estimate that 6% to 62% of the variance in reporting different types of trauma is attributable to genetics (7, 8, 9, 10, 11, 12). Interpersonal assaultive traumas (e.g., physical and sexual assault) have higher heritability than noninterpersonal or nonassaultive traumas (e.g., accidents) (7,9,12). In relation to these observations, stressful life events dependent on one’s behavior (e.g., fights) are more heritable than events that are independent of an individual's own behavior (e.g., natural disasters), with the latter occurring more often due to chance (5, 13). However, being at higher genetic risk for reported trauma does not signify that an individual is genetically predestined to experience trauma. Furthermore, a large proportion of the total phenotypic variability of reported trauma is not attributable to genetics. The environment itself may be harmful, or a perpetrator may exploit individuals in vulnerable circumstances (11,14). However, environmental risk factors are generally unstable, idiosyncratic, and thus, unpredictable and challenging to examine (15). Exploring traits genetically related to reported trauma in different environmental contexts may provide a framework for social research to help determine trauma risk factors and protect vulnerable individuals (6).

Heritable behavioral characteristics may contribute to the likelihood of experiencing certain events. Personality traits, such as openness to experience and antisocial behavior, are phenotypically and genetically correlated with reporting interpersonal assaultive trauma (16). Such partially heritable characteristics may contribute to the heritability of reported trauma through gene-environment correlation (rGE), whereby the environment reflects an individual’s genetic propensities via 3 different processes (17). Passive rGE occurs when a relative’s genotype, such as parental genetic variation contributing to risk-taking behaviors, shapes the child’s environment and potentially creates an unsafe home (17, 18, 19). The environment that the parent creates and the parental genotype are correlated, as the child receives both from their biological parents. Thus, parental environmental effects may be captured in genetic analyses of offspring traits (17). Evocative rGE arises when an individual’s genotype shapes how others engage with them. For example, a child’s behavioral difficulties may evoke verbal and physical discipline due to the carer’s expectations of how a child should behave (17). Active rGE involves an individual’s genetic disposition to, for example, risk-taking modifying and selecting their environment (17,20), leading to differing risks of exposure to adverse environments.

Correlations between genetic factors and retrospective reports of trauma may also in part be driven by heritable characteristics influencing the subjective interpretation, willingness to disclose, and recollection of events (21,22). Genetic research has largely relied on retrospective self-reports of trauma exposure, which may be more susceptible to genetically influenced perceptions and recollection of events, as opposed to more objective measures prospectively recorded closer to the time of exposure (e.g., court records, caregiver reports) (22). Memory, emotional regulation, and interpretation biases are partly heritable (23, 24, 25, 26) and are associated with retrospective reporting of trauma in early life (27). Individual differences in subjective experiences are likely partly influenced by genetics (28,29). Subjective appraisal of trauma is important for posttraumatic psychopathology, which is more strongly associated with retrospective self-reports of trauma than objective court records (30). Individual, partially heritable differences in personality traits such as neuroticism and agreeableness may explain the discrepancy between retrospective and prospective measures of trauma (30, 31, 32). Furthermore, the consistency and frequency of self-reports are impacted by individual factors involved in the willingness to disclose a traumatic event, such as perception of stigma, fear of negative consequences, or preexisting relationships with the perpetrator (33, 34, 35, 36). Lack of disclosure is a barrier to therapeutic and legal interventions (35). Thus, a better understanding of the heritable factors that impact the retrospective report of trauma experiences could help improve posttraumatic support.

In sum, the influences on retrospectively reported trauma are complex and difficult to disentangle. A range of heritable traits may be involved. Heritability and genetic correlations between traits can be estimated using genome-wide association study (GWAS) summary statistics (37, 38). The proportion of heritability explained by common genetic variants (single nucleotide polymorphism–based heritability [h^2^_SNP_]) ranges from 6% to 9% for reported interpersonal trauma during childhood (20,39) to 18% during childhood and adulthood combined (40). This accounts for a large proportion of the reported twin heritabilities estimated at 20% to 62% (7,9, 10, 11, 12). Reported trauma shows genetic correlations with psychiatric disorders, current mental state, personality traits, lifestyle factors, and sociodemographic traits (20,39,40). However, these studies did not analytically explain the extent to which the h^2^_SNP_ of reported traumas reflects genetic correlations with these complex traits. Identifying specific traits that explain a large proportion of h^2^_SNP_ can guide follow-up analyses in assessing certain characteristics involved in rGE and/or the subjective experience of trauma.

We hypothesized that genetic variants associated with relevant behavioral and cognitive traits would overlap with those associated with reported trauma, such that no residual genetic variance of reported trauma would remain after accounting for genetically correlated traits. Twin studies have used multivariate structural equation modeling (SEM) to examine the residual genetic variance of life event measures after accounting for genetically correlated traits (41,42). To our knowledge, multivariate SEM has not been used to explore the extent to which specific heritable characteristics capture the heritability of reported trauma. Twin studies are limited in assessing only a moderate number of traits and environmental measures in the same individuals, which may be particularly challenging in the case of more severe environmental exposures such as trauma (43). In contrast, the multivariate SEM extension to GWAS summary statistics (44) allows the inclusion of many more traits measured in different individuals. Here, we decompose h^2^_SNP_ of reported trauma using genomic multiple regression with the Genomic SEM R package (44). Our primary aim was to measure the amount of residual genetic variance of reported trauma that remains after accounting for genetically correlated traits. Our secondary aim was to identify the traits contributing to h^2^_SNP_ of reported trauma from hundreds of complex traits that were systematically assessed.

## Methods and Materials

### Samples and Measures

We used summary statistics from the largest published GWAS of reported trauma as of 2021 on childhood maltreatment (20). This GWAS built on our previous work (40), extending it to assess childhood maltreatment specifically, and included 185,414 participants predominantly of European genetic ancestry from 5 datasets: UK Biobank (45), ABCD (Adolescent Brain Cognitive Development) Study (46), ALSPAC (Avon Longitudinal Study of Parents and Children) (47,48), Generation R (49,50), and PGC (Psychiatric Genomics Consortium) (39). Childhood maltreatment was defined as reports of emotional, sexual, and physical abuse and emotional and physical neglect. Most traumas (91.5%) were retrospectively self-reported (*n* = 169,766); however, a small proportion (8.5%) were reported prospectively by a parent or caregiver (*n* = 15,651). The genetic correlation between retrospective and prospective childhood maltreatment was previously reported as 0.72 (SE = 0.36; *p* = .05) (20). Further methodological details can be found elsewhere (20). In the original publication, h^2^_SNP_ of the continuous meta-analyzed phenotype of childhood maltreatment was 0.08 (SE = 0.01) using linkage disequilibrium (LD) score regression (20). We also analyzed GWAS summary statistics from our previous study (40) of a retrospectively reported lifetime trauma phenotype that more broadly captures trauma occurring in both childhood and adulthood in the UK Biobank (45) (Supplemental Methods in Supplement 1).

### Bivariate Genetic Correlations

To identify traits associated with the genetic component of reported trauma, we used bivariate LD score regression (51,52) to measure the genetic correlations (*r*_g_) between reported trauma and a wide range of complex traits. We tested 576 traits from GWAS summary statistics for *r*_g_ with reported trauma. We excluded the major histocompatibility complex region from our analyses (51,53). We considered traits for downstream analyses in Genomic SEM if they met the following criteria: |*r*_g_| with reported trauma of > 0.25 and |*z*| statistic ≥ 5, GWAS mean χ^2^ value > 1.02, and h^2^_SNP_ *z* statistic ≥ 5. These thresholds were based on recommendations by the software developers (54,55). All traits that met these criteria were also statistically significant after Bonferroni correction for multiple testing (α = 0.05/number of traits; *p* ≤ 8.68  × 10^−5^), which was less stringent than our selected threshold *r*_g_ |*z*| statistic ≥ 5 (equivalent to *p* ≤ 5.73 × 10^−7^). The criteria were stringent to restrict the number of traits included in downstream analyses to well-powered GWASs with potentially larger genetic contributions to h^2^_SNP_ of reported trauma.

### Genomic SEM

To decompose h^2^_SNP_ of reported trauma, we used the Genomic SEM R package version 0.0.5 (https://github.com/MichelNivard/GenomicSEM/wiki) (44). Genomic SEM is a multivariate extension of LD score that constructs covariance matrices from h^2^_SNP_ and *r*_g_ calculated by LD score. GWAS samples can overlap for Genomic SEM as the sampling covariance matrices adjust for potential sample overlap. All GWAS summary statistics in our analyses were based on individuals drawn from European genetic ancestries.

We fitted fully saturated genomic multiple regression models. This approach simultaneously regressed the outcome (i.e., reported childhood maltreatment) on various explanatory variables, which modeled genetic correlations between each explanatory variable. This was informative in two ways. First, we estimated the residual genetic variance of reported trauma not explained by the genetics of the explanatory variables. Second, we estimated the unique contribution of each explanatory variable to the genetic component of reported trauma independent of other explanatory variables (i.e., conditional genetic association, termed b_*g*_). We selected explanatory variables based on results from bivariate LD score regression. We introduced one explanatory variable at a time, iteratively, from the most highly correlated trait with reported trauma to the least correlated trait. For all models, we used the default diagonally weighted least squares estimator, in which the precision of genetic covariances (e.g., due to GWAS sample size) is considered.

We identified a practical limit of ≤11 explanatory variables in a fully saturated multiple regression model. Standard errors increased as more explanatory variables were added (>11 explanatory variables resulted in standard errors > 1). Therefore, we had to fit 2 models for categorically distinct traits identified in LD score regression analysis: 1) health and behavioral traits and 2) psychiatric disorders.

## Results

Results from analyzing reported childhood maltreatment were highly similar to that of reported lifetime trauma. We therefore present results for the more highly powered GWAS of childhood maltreatment (power quantified by h^2^_SNP_ *z* statistic = 18.7; mean χ^2^ value = 1.27) and report our findings for reported lifetime trauma (h^2^_SNP_ *z* statistic = 15.6; mean χ^2^ value = 1.22) in the Supplemental Results in Supplement 1 and Tables S7 through S11 in Supplement 2.

### Traits Genetically Correlated With Reported Childhood Maltreatment

**Figure 1:**
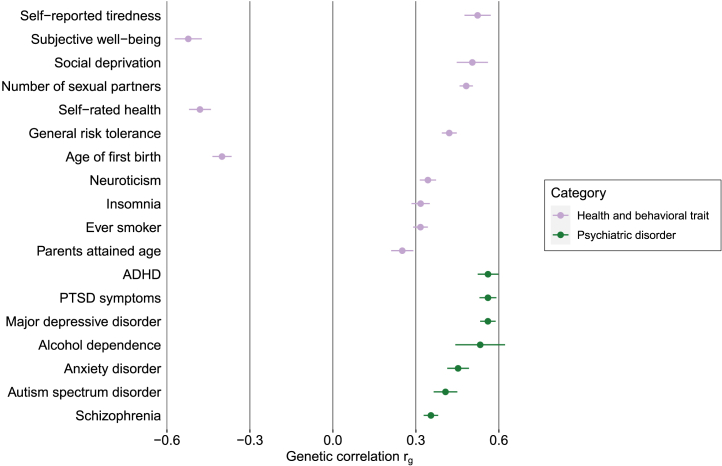
Top bivariate genetic correlations (*r*_g_) between reported childhood maltreatment and various heritable traits. We calculated the correlations using linkage disequilibrium score regression. We tested correlations with 576 traits. Only traits with an |*r*_g_| > 0.25 and *r*_g_ |*z*| statistic ≥ 5, sufficiently powered with a mean χ^2^ value > 1.02 and a common genetic variant–based heritability *z* statistic ≥ 5 are shown. Bars represent standard errors. ADHD, attention-deficit/hyperactivity disorder; PTSD, posttraumatic stress disorder.

Figure 1 shows the 18 bivariate genetic correlations with childhood maltreatment (|*r*_g_| > 0.25 and *r*_g_ |*z*| statistic ≥ 5). Genetic correlations with all 576 traits are summarized in Table S1 in Supplement 2. After filtering for sufficiently powered GWASs, 18 traits were genetically correlated with childhood maltreatment with |*r*_g_| > 0.25 and *r*_g_ |*z*| statistic ≥ 5 (Figure 1; Table S2 in Supplement 2). The pairwise genetic correlations among the health and behavioral traits and psychiatric disorders modeled in Genomic SEM are shown in Tables S3L and S4H in Supplement 2, respectively. The most well-powered GWAS was retained in cases where pairwise |*r*_g_| in each category was not significantly different from 1 (calculated using the χ^2^ distribution function and [(|*r*_g_| − 1)/SE]^2^ in R version 4.1.1) (40,56).

### Genomic Multiple Regression

#### Health and Behavioral Traits

**Figure 2:**
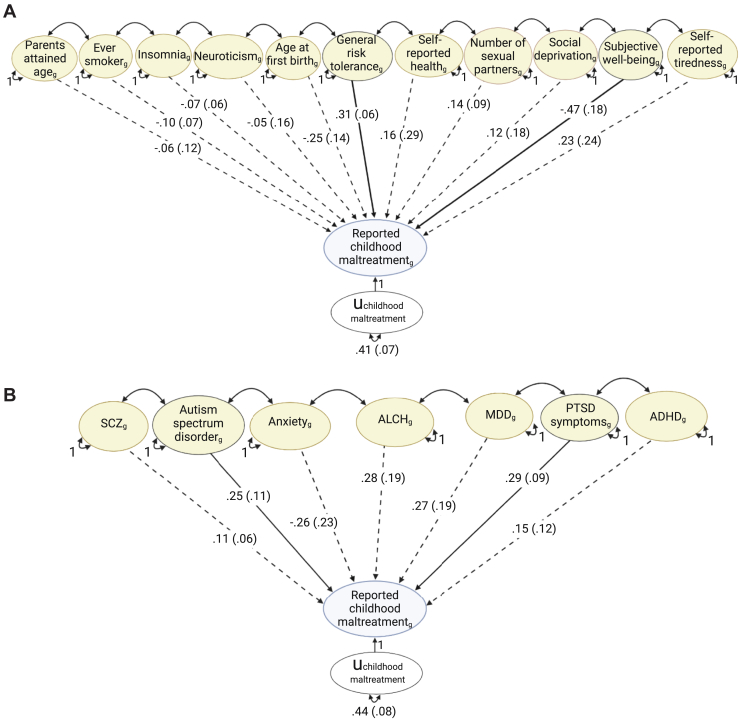
Path diagrams representing results from genomic multiple regression analyses. Models were specified in Genomic SEM using the weighted least squares estimator. The genetic components of **(A)** 11 health and behavioral traits and **(B)** 9 psychiatric disorders are the simultaneously fitted explanatory variables of the genetic component of reported childhood maltreatment. Single-headed arrows are conditional genetic associations (b_*g*_ ± SE) between the explanatory variables and childhood maltreatment independent of the genetic influences of the other explanatory variables. A solid line indicates that the conditional genetic association is significant, and a dashed line indicates the conditional genetic association is nonsignificant. Double-headed arrows connecting explanatory variables represent genetic correlations; for simplicity, these values are not shown here but are in Tables S3L and S4H in Supplement 2. Double-headed arrows connecting the genetic component of childhood maltreatment to itself is the residual genetic variance of childhood maltreatment (u_maltreatment_ ± SE) that is unexplained by the genetic influence of either the psychiatric disorders or the health and behavioral traits. ADHD, attention-deficit/hyperactivity disorder; ALCH, alcohol dependence; MDD, major depressive disorder; PTSD, posttraumatic stress disorder; SCZ, schizophrenia.

As noted above, as we were able to include a maximum of 11 explanatory variables at once, we specified separate models for health and behavioral traits and psychiatric disorders. The path diagram in Figure 2A shows the results for 11 health and behavioral traits simultaneously specified as explanatory variables of the genetic component of childhood maltreatment. Heritability can be defined as the proportion of variance between individuals for a given trait or disorder that is accounted for by genetic factors. The residual genetic variance is the amount of heritable variation of childhood maltreatment that is unexplained by the genetic factors of the explanatory variables in the model. The genetic influences on these health and behavioral traits explained 59% of h^2^_SNP_ of childhood maltreatment (1 minus the residual genetic variance of 0.41 ± 0.07; *p* = 7.76 × 10^−9^).

When controlling for the genetic influences of the other explanatory variables, the majority of conditional genetic associations between reported childhood maltreatment and health and behavioral traits were nonsignificant (*p* > .05) (dashed lines in Figure 2A and Table S3K in Supplement 2). Therefore, most of the genetic correlations between childhood maltreatment and health and behavioral traits were shared with the other health and behavioral traits in the model. The shared genetic component across these traits may drive the direction of some bivariate genetic correlations, as several traits showed conditional associations with childhood maltreatment in the opposite direction (e.g., reported health *r*_g_ = −0.48; b_*g*_ = 0.16). However, 2 traits were uniquely genetically associated with childhood maltreatment, over and above their genetic correlations with the other traits: subjective well-being (b_*g*_ = −0.47 ± 0.18; *p* = .01) and general risk tolerance (self-report of how willing the individual is with taking risks; b_*g*_ = 0.31 ± 0.06; *p* = 9.96 × 10^−7^). Models where each health trait was introduced iteratively are shown in Table S3A–K in Supplement 2.

#### Psychiatric Disorders

When taking into account the genetic influences of all 9 psychiatric disorders in a genomic multiple regression model (Figure 2B), the residual genetic variance of reported childhood maltreatment was 0.44 ± 0.08 (*p* = 9.23 × 10^−9^). Thus, 56% (calculated as 1 − 0.44) of h^2^_SNP_ of childhood maltreatment was explained by genetic components of these psychiatric disorders. In this model, most psychiatric disorders shared their genetic overlap with childhood maltreatment, as indicated by nonsignificant conditional associations after accounting for shared genetics (*p* > .05) (Figure 2B; Table S4G in Supplement 2). Autism spectrum disorder (ASD) (b_*g*_ = 0.25 ± 0.11, *p* = .02) and posttraumatic stress disorder (PTSD) symptoms (b_*g*_ = 0.29 ± 0.09, *p* = 1.40 × 10^−3^) had significant conditional associations with childhood maltreatment independent of other disorders. Table S4A–G in Supplement 2 shows genomic multiple regression results when psychiatric disorders were included in iterative stages.

### Model of Independently Contributing Health and Psychiatric Traits

To explore the contribution of the independently genetically associated traits (i.e., ASD, PTSD, general risk tolerance, and subjective well-being), as a sensitivity analysis, we specified the genetic component of these 4 traits as explanatory variables of the genetic component of reported childhood maltreatment simultaneously (Figure S1 in Supplement 1; Table S5 in Supplement 2). The residual genetic variance of reported childhood maltreatment was 0.42 ± 0.06 (*p* = 1.24 × 10^−12^). This means 58% of h^2^_SNP_ of childhood maltreatment was explained by the genetic component of ASD, PTSD, general risk tolerance, and subjective well-being. In a model with 2 additional traits (attention-deficit/hyperactivity disorder and self-reported tiredness), the residual genetic variance of childhood maltreatment decreased by only approximately 2% (0.40 ± 0.06; *p* = 2.11 × 10^−12^) (Supplemental Results in Supplement 1; Table S6 in Supplement 2).

## Discussion

Using genomic multiple regression in Genomic SEM, we identified traits that accounted for approximately 60% of h^2^_SNP_ of reported trauma. Health and behavioral traits together accounted for 59% of h^2^_SNP_ variance, while the model exploring psychiatric disorders explained 56%. In both models, a shared genetic component was observed across traits except for subjective well-being, general risk tolerance, PTSD symptoms, and ASD, which were independently associated with childhood maltreatment. Together, these latter 4 traits alone were sufficient to explain 58% of h^2^_SNP_ of reported childhood maltreatment.

We found similar results for retrospective lifetime trauma, which included adult trauma. We could not directly compare adulthood and childhood trauma as the lifetime trauma phenotype included both measures. Our findings suggest that the exact timing of trauma does not strongly affect the proportion of genetic variance accounted for by health and psychiatric traits. However, replication in nonoverlapping datasets and appropriate trauma measures are required to make strong conclusions about differences between adulthood and childhood trauma.

Independently associated traits, in addition to the genetic components shared across health and psychiatric traits, may be involved in rGE and/or the reporting of such environments as traumatic. This raises the question of which processes explain these associations and could inform strategies to minimize risk and consequences of trauma.

General risk tolerance is measured by endorsing a willingness to take risks, broadly capturing risk-taking behaviors (57). rGE may explain the genetic contribution of risk tolerance to the genetic component of reported trauma, consistent with a previous study that found environmental adversities mediate the association between genetic propensity for risk taking and reported childhood maltreatment (22). A child may passively inherit a parent’s genetic propensity for risk-taking behaviors, such as substance use, and be exposed to an environment where the child may be neglected or abused (19,22). An individual’s own genetic propensity for risk taking may increase exposure to potentially adverse environments (16,20,58). However, active rGE may be less prominent during childhood than passive rGE and therefore a less plausible explanation for our findings (59). Alternatively, risk tolerance may capture behaviors associated with disclosure of trauma. A barrier to disclosing trauma includes perceiving it as a risk and fearing negative consequences (33). As such, individuals who take fewer risks may also be less inclined to disclose traumas. Further research is required to elucidate the mechanisms that explain the genetic association between general risk tolerance and reported trauma.

Subjective well-being captures an individual’s cognitive evaluation of life satisfaction and positive affectivity (60,61). Our findings may reflect the role of such cognitions in the perception, recall, and reporting of trauma exposure. As found with life events, individuals with a genetic propensity to positive subjective well-being may be less likely to report trauma retrospectively, while the converse may occur with negative subjective well-being (29). A positive disposition could bias toward a more positive recall of experiences (31,32). This could explain why some individuals with objective records of experiencing childhood maltreatment do not retrospectively self-report maltreatment (30). Such individuals are also less likely to develop posttraumatic psychopathology (30). Positive affectivity may contribute to resiliency by countering the adverse effects of stressful experiences due to efficient regulation of negative emotions (62). Conversely, if negative affectivity indicates greater sensitivity to trauma and vulnerability to psychopathology, this could have implications for screening for psychopathology risk following trauma (63).

The independent genetic association between ASD and childhood maltreatment is supported by several previous studies (22,64,65). Family-based polygenic score analyses exploring genetic differences between siblings suggest that a greater risk of childhood maltreatment in individuals with ASD is partly attributable to evocative and active rGE (20). Difficulty processing social cues may place an individual at greater risk of harmful environmental situations, such as exploitation by a perpetrator (64,66). Furthermore, individuals with ASD may experience a broader range of life experiences as traumatic (67). An association between the polygenic score for ASD and trauma has been consistently found with retrospectively, but not prospectively, reported trauma (22,64,65,68, 69). One study found the association between a polygenic score for ASD and retrospectively reported trauma was independent of rGE (22). Together, these findings suggest the importance of subjective trauma interpretation in ASD. Future research should determine the specific heritable components of ASD related to the subjective experience of and exposure to trauma and the potential for screening for posttraumatic symptoms in ASD to provide appropriate support (67).

As trauma exposure is necessary for a PTSD diagnosis, the unique genetic association between reported trauma and PTSD symptoms could be explained by trauma exposure increasing the risk of PTSD. However, there are plausible reverse or bidirectional mechanisms. This includes passive rGE, whereby parental genetic predisposition to PTSD may act to increase the risk of trauma exposure in the child, as suggested by studies of PTSD and parenting (70). However, evidence suggests the association between a higher genetic risk for PTSD and increased self-report of childhood trauma could be explained by subjective interpretation processes and not rGE (22). This is supported by previous findings that PTSD polygenic scores are not associated with objectively assessed trauma exposure severity (71). Except for ASD and PTSD, genetic associations between reported childhood maltreatment and the other psychiatric disorders were explained by genetic factors shared across all other psychiatric disorders included in the model. However, GWASs often use brief phenotypic measures to achieve sufficient power, which may impact our ability to detect disorder-specific genetic influences (72). Shared genetics may underlie transdiagnostic psychological mechanisms, such as those involved in the subjective experience of trauma, which is more robustly associated with psychiatric disorders than objective measures of trauma (30). Our findings support targeting transdiagnostic pathways to reduce the general risk of psychopathology following trauma (73,74).

Our modeling approach has several limitations. First, the model is fully saturated, and we could not objectively estimate which model best fits the data. Second, we were limited by the number of traits that could be fitted in one model. However, sensitivity analyses did not indicate that all of the residual genetic variance of childhood maltreatment could be explained if all available genetically correlated traits were accounted for in one model. Third, our estimates are based on lower bounds of the total genetic variance explained by common genetic variants that can be, as captured by h^2^_SNP_, estimated from summary statistics and may differ from results using more advanced methods or individual-level genetic data (40,75).

Further research is needed to establish the role of traits in terms of whether they are associated with the risk of exposure or the interpretation and recollection of events. Preliminary evidence suggests that h^2^_SNP_ of prospective trauma is lower than retrospectively reported trauma (20). Thus, genetic factors involved in the retrospective reporting of trauma may have a greater impact on h^2^_SNP_ of trauma than traits involved in the exposure of events. The residual h^2^_SNP_ of trauma may reflect traits involved in memory recall (27,32) that lack adequately powered GWASs. Alternatively, unaccounted for parental traits involved in passive rGE, such as parental antisocial behavior contributing to an unsafe environment, may partly explain the residual genetic variance (17). Disentangling the genetic associations with vulnerability to exposure and those with the subjective experience of trauma will be important for distinguishing whether factors are relevant to trauma prevention or posttraumatic interventions.

In summary, we systematically examined traits genetically correlated with reported trauma, implicating possible mechanisms that partly explain h^2^_SNP_ of trauma. Potentially, indirect genetic effects regulating behavior and cognition are associated with trauma exposure and/or retrospectively self-reporting trauma. We emphasize that our findings do not suggest that an individual is ever at fault or responsible for their exposure to trauma. Furthermore, h^2^_SNP_ of reported trauma does not mean that some individuals are genetically determined to experience trauma. Most of the phenotypic variance of reported trauma is explained by the environment, which is malleable and can be modified into a more supportive and protective environment to mitigate vulnerabilities. For example, if a genetic propensity for ASD, PTSD, general risk tolerance, and subjective well-being reflect genetic risk to reported trauma, more social support may protect such individuals and alleviate adverse posttraumatic effects. However, our findings are correlational, not necessarily causal, and better delineation of the processes involved is needed. Future studies could assess the specific role of these traits in large family-based datasets using within-family designs (43). Disentangling passive from evocative and active rGE that may explain our trait-specific associations could have implications for prevention strategies. As GWASs increase in power, the direction of causal relationships or testing the types of pleiotropy could be explored through Mendelian randomization techniques (20,76). Such approaches are crucial to understanding vulnerability to trauma exposure and the subjective interpretation of trauma.

## Supplementary Material

Supplementary Data

Key Resources Table
