# Supplementary material for: Genetic Decomposition of the Heritable Component of Reported Childhood Maltreatment
Source: Biol Psychiatry Glob Open Sci. 2023 Mar 24;3(4):716–24. doi: 10.1016/j.bpsgos.2023.03.003 (PMC10593925; doi:10.1016/j.bpsgos.2023.03.003)
Supplement: Supplementary Data [file mmc1.pdf]

SUPPLEMENTARY INFORMATION

Genetic Decomposition of the Heritable Component of Reported Childhood Maltreatment

ter Kuile *et al.*

Supplementary methods

<u>Sample and measures</u>

Self-reported lifetime trauma in the UK Biobank

We also analysed published GWAS summary statistics of a lifetime trauma phenotype that more broadly captures trauma occurring in both childhood and adulthood in the UK Biobank (1,2). The lifetime trauma GWAS included 35,269 individuals who retrospectively reported trauma and 63,451 individuals who reported no traumas (1). Participants were predominantly of European ancestry and answered the online mental health questionnaire (3). Participants were considered reporters of trauma if they self-reported at least two types of lifetime trauma and were excluded from analyses if they only reported one. These trauma types were selected based on their association in the UK Biobank with major depressive disorder (MDD) and needed to show an odds ratio >2.5 for selection. These pre-selected self-reported traumas occurred in childhood (i.e., felt loved less than often, felt hated by a family member, sexually abused), adulthood (i.e., partner physical violence, belittlement, and sexual interference) and one-lifetime item (i.e., victim of sexual assault). Individuals were considered non-reporters of trauma if they did not self-report any pre-selected traumas. Further methodological details can be found in the original publication (1). The h^2_{SNP} of this self-reported lifetime trauma definition using LDSC was 0.18 (s.e = 0.01) on the liability scale

(1). We also calculated the genetic correlation between reported childhood maltreatment and retrospectively reported lifetime trauma, and tested if the genetic correlation was significantly different from 1 (calculated using the chi-squared distribution function and $[(|r_g|-1)/se]^2$ in R v. 4.1.1) (1,4).

Supplementary results

Sensitivity analyses

Model of independently contributing health and psychiatric traits

All four traits had significant conditional associations with reported childhood maltreatment independent of the other traits; subjective well-being (b_g = -0.32 ± 0.08; $P = 1.22 \times 10^{-4}$), general risk tolerance (b_g = 0.36 ± 0.04; $P = 9.15 \times 10^{-21}$), PTSD symptoms (b_g = 0.32 ± 0.05; $P = 7.98 \times 10^{-10}$) and ASD (b_g = 0.21 ± 0.06; $P = 5.80 \times 10^{-4}$). The residual genetic variance of reported childhood maltreatment was 0.42 ± 0.06 ($P = 1.24 \times 10^{-12}$; **Supplementary Figure 1**). Direct estimation of the overlap in the residual genetic variance of childhood maltreatment that remained in the psychiatric disorder model and the health trait model was not possible. To address this, we specified a genomic multiple regression model that included the four independently contributing traits and two additional traits. These two additional traits broadly capture the shared genetic associations observed across the psychiatric disorders (ADHD) and health and behavioural traits (self-reported tiredness). In this sensitivity model, the residual genetic variance of childhood maltreatment was 0.40 ± 0.06 ($P = 2.11 \times 10^{-12}$), explaining 60% of the h^2_{SNP} of childhood maltreatment (**Supplementary Table 6**). The addition of these traits made only a ~2% difference in the residual genetic variance of childhood maltreatment. Thus, if all psychiatric, health, and behavioural traits could be accounted for in one model, it is unlikely this would greatly reduce

the h^2_{SNP} of childhood maltreatment. This means that 40-42% of the h^2_{SNP} of childhood maltreatment remains unexplained by the genetic components of traits included in our models.

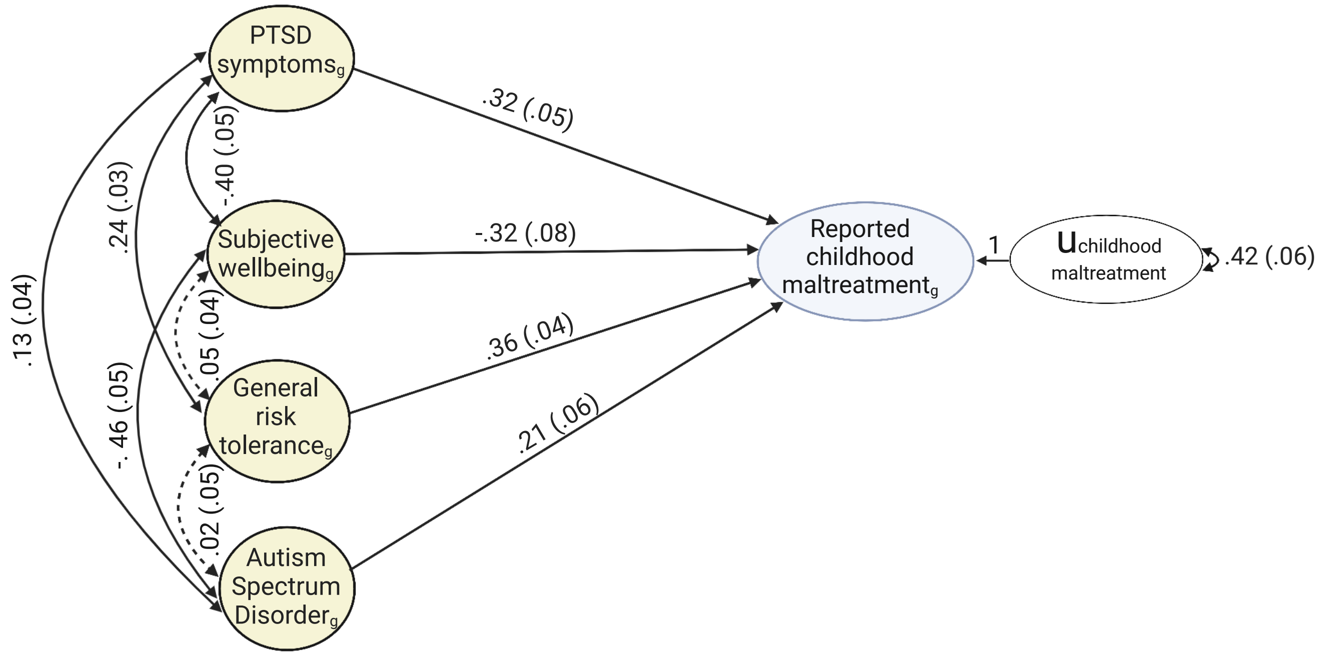

Supplementary Figure 1. Path diagram representing results from genomic multiple regression analyses of independently contributing traits to the genetic component of childhood maltreatment. *Autism spectrum disorder (ASD), post-traumatic stress disorder (PTSD) symptoms, general risk tolerance, and subjective well-being were selected based on results shown in **Figures 2A** and **2B**. A genomic multiple regression was specified in Genomic SEM. We regressed the genetic component of childhood maltreatment on the genetic components of four independently genetically associated traits. We used a weighted least-squares estimator. Single-headed arrows are conditional genetic associations (b_g ± SE) between the explanatory variables and childhood maltreatment independent of the genetic influences of the other explanatory variables. A solid line indicates that the conditional genetic association is significant. Double-headed arrows connecting explanatory variables represent genetic correlations (r_g ± SE). Double-headed arrows connecting the genetic component of childhood maltreatment to itself is the residual genetic variance of childhood maltreatment ($u_{maltreatment}$ ± SE) that is unexplained by the genetic influence of the explanatory variables.*

Self-reported lifetime trauma

The genetic correlation between reported childhood maltreatment and retrospectively reported lifetime trauma was high (r_g = 0.884 ± 0.04; P = 1.00 × 10^{-122}), but significantly different from 1 (P = 0.0211). Results for lifetime trauma are in **Supplementary Tables 7-11**. We identified 18 traits with significant genetic correlations (r_g >±.25) with retrospective lifetime trauma after Bonferroni correction for multiple testing. Most of these traits were also top genetic correlations with childhood maltreatment and categorised into the same two groups of psychiatric disorders and health and behavioural traits. In genomic multiple regression analyses, the residual genetic variance of lifetime trauma was 0.25 ±0.13 (P = 0.059) and 0.47 ±0.07 (P = 8.00 × 10^{-11}) after taking into account the genetic associations with health and behavioural traits and psychiatric disorders, respectively. In these models, independent genetic associations with lifetime trauma were identified for ASD (b_g = 0.36 ± 0.10, P = 2.28 × 10^{-4}), PTSD symptoms (b_g = 0.28 ± 0.09; P = 1.13 × 10^{-3}), subjective well-being (b_g = -0.67 ± 0.33; P = 0.048) and general risk tolerance (b_g = 0.42 ± 0.10; P = 4.29 × 10^{-5}). When these four traits were included in one genomic multiple regression model, the residual genetic variance of lifetime trauma was 0.33 ±0.07 (P = 1.46 × 10^{-6}). Given the consistency in findings between retrospectively reported lifetime trauma and reported childhood maltreatment, this suggests that the inclusion of a small prospective sample in the reported childhood maltreatment meta-analysis did not impact our results. Our findings likely reflect associations with retrospectively reported and not prospectively reported childhood maltreatment, given the lack of power of the prospective GWAS (h^2_{SNP} Z score = 1.39) (5).

Supplementary references

1. Coleman JRI, Peyrot WJ, Purves KL, Davis KAS, Rayner C, Choi SW, *et al.* (2020): Genome-wide gene-environment analyses of major depressive disorder and reported lifetime traumatic experiences in UK Biobank. *Mol Psychiatry*. https://doi.org/10.1038/s41380-019-0546-6
2. Sudlow C, Gallacher J, Allen N, Beral V, Burton P, Danesh J, *et al.* (2015): UK biobank: an open access resource for identifying the causes of a wide range of complex diseases of middle and old age. *PLoS Med* 12: e1001779.
3. Davis KAS, Coleman JRI, Adams M, Allen N, Breen G, Cullen B, *et al.* (2020): Mental health in UK Biobank - development, implementation and results from an online questionnaire completed by 157 366 participants: a reanalysis. *BJPsych Open* 6: e18.
4. Team RC (n.d.): R Core Team (2019) RA Language and Environment for Statistical Computing. R Foundation for Statistical Computing, Vienna, Austria. *References-Scientific Research Publishing*.
5. Warrier V, Kwong ASF, Luo M, Dalvie S, Croft J, Sallis HM, *et al.* (2021): Gene–environment correlations and causal effects of childhood maltreatment on physical and mental health: a genetically informed approach. *The Lancet Psychiatry* 8: 373–386.
